# A co-creation approach to selecting and adapting adolescent tobacco and air-pollution interventions and strategies for implementation in diverse countries: insights from FRESHAIR4Life

**DOI:** 10.1186/s43058-026-01044-4

**Published:** 2026-07-20

**Authors:** Purva Abhyankar, Alexander Lepe, Jurjen van der Schans, Floor A. van den Brand, Faraz Siddiqui, Saima Saeed, Javeria Akhter, Amina Khan, Mian Ali Kamran Baba, Ioanna Tsiligianni, Izolde Bouloukaki, Antonios Christodoulakis, Winceslaus Katagira, Bruce Kirenga, Cristina Isar, Adriana Antohe, Anca Lupu, Andrea Neculau, Talant Sooronbaev, Gulzada Mirzalieva, Aijan Taalaibekova, Rianne van der Kleij, D. K. Arvind, Siân Williams, Luís Carvalho, Hilary Pinnock

**Affiliations:** 1 Usher Institute, University of Edinburgh, 5‒7 Little France Road, Edinburgh BioQuarter ‒ Gate 3, Edinburgh, UK; 2 Global Health Unit, Department of Health Sciences, University Medical Center Groningen, University of Groningen, Groningen, The Netherlands; 3 Socio-Economic Panel, German Institute for Economic Research, Berlin, Germany; 4 Department of Family Medicine (CAPHRI), Faculty of Health, Medicine and Life Sciences, Maastricht University, Maastricht, The Netherlands; 5 Department of Health Sciences, University of York, York, UK; 6 Indus Hospital and Health Network, Karachi, Pakistan; 7 The Initiative, Islamabad, Pakistan; 8 Department of Social Medicine, Faculty of Medicine, University of Crete, Heraklion, Greece; 9 College of Health Sciences, Makerere University Lung Institute, Makerere University, Kampala, Uganda; 10 Centrul National de Studii pentru Medicina Familiei (CNSMF RespiRO), Bucharest, Romania; 11 Universitatea Transilvania, Brasov, Romania; 12 Department of Pulmonology and Allergology, National Center of Cardiology and Internal Medicine named after academician M.Mirrakhimov, Bishkek, Kyrgyzstan; 13 Leiden University Medical Center, Public Health & Primary Care, Albinusdreef 2, Leiden, 2333 ZA The Netherlands; 14 Centre for Speckled Computing, School of Informatics, University of Edinburgh, Edinburgh, UK; 15 International Primary Care Respiratory Group (IPCRG), Edinburgh, UK

**Keywords:** Tobacco use, Nicotine use, Air pollution, Implementation research, Intervention selection, Adaptation, Co-creation, NCD prevention, Stakeholder involvement

## Abstract

**Background:**

Evidence-based interventions to reduce adolescents’ exposure to tobacco and air pollution are rarely implemented in low- and middle-income countries, often because they lack contextual fit. The ‘FRESHAIR4Life’ project aimed to optimise implementation of contextually adapted, evidence-based interventions in Greece, Kyrgyzstan, Pakistan, Romania, and Uganda. Selecting appropriate interventions required deliberate, inclusive, and context-sensitive decision-making. This paper outlines the co-creation approach for selecting and adapting interventions, developing implementation strategies and addressing the challenges that arose.

**Methods:**

Each country followed a four-step process. First, findings from local situational analyses were consolidated to identify barriers and facilitators across individual, community, and system levels and to map targets for change using the Theoretical Domains Framework. This was followed by a workshop with country-teams to identify key risk factors, target behaviours, relevant populations and settings, and to shortlist potential interventions from the FRESHAIR4Life palette alongside implementation strategies using option grids and ranking. A subsequent stakeholder workshop applied a Socio Technical Allocation of Resources informed approach to elicit perspectives on the interventions’ value using qualitative (Conversation Café style discussions) and quantitative methods (ratings of need, importance, likely reach, feasibility, and local adaptations required, fixed budget allocation exercises or ranking of options). Finally, selection meetings with country teams finalised intervention choices and implementation strategies, informed by stakeholder input.

**Results:**

Countries chose to target the risk factors that were considered most prevalent and the biggest threat to adolescent health in their settings. Of the four countries addressing tobacco use, Pakistan, Greece, and Romania selected ‘implementation intentions (If-Then approach)’ for prevention, while Kyrgyzstan selected ‘very brief advice plus behavioural counselling (VBA+)’ for cessation. All three countries addressing air pollution, Pakistan, Uganda and Kyrgyzstan selected social media campaigns. Key challenges included: (1) establishing a sound basis for selection decisions, which was overcome by adopting a systematic, transparent and inclusive decision process (2) ethical and practical considerations for addressing tobacco/nicotine use within school-based prevention, tackled by adapting intervention to include offer of cessation support (3) limited evidence-based interventions targeting ambient air pollution, addressed by flexibly moving beyond the palette (4) the evolving nature of implementation strategies and (5) embedding interventions into routine practice, both addressed through flexibility and adaptation.

**Conclusion:**

This study shows that, across diverse country contexts, defensible intervention selection depends not only on which intervention is chosen, but on how choices are made and by whom. A transparent, stakeholder-owned co-creation process, involving the target audience of adolescents, helped country teams navigate trade-offs between evidence, local burden, feasibility, fit with routine practice, and ethical obligations, while exposing important evidence gaps for individual- and community-level ambient air-pollution interventions in LMICs.

**Supplementary Information:**

The online version contains supplementary material available at https://doi.org/10.1186/s43058-026-01044-4.

Contributions to the literature

- Demonstrates a structured, stakeholder-owned co-creation process for selecting and adapting interventions in diverse contexts, where evidence is often limited.
- Shows how systematic, transparent decision-making can help prioritise intervention and implementation strategy choices in complex, resource-constrained settings.
- Highlights how co-creation can reconcile tensions between evidence-based interventions and contextual fit, informing defensible, context-appropriate decisions.
- Identifies the challenges and suggests an approach to identifying evidence-based interventions that could be flexibly and context-sensitively adapted to target young people in diverse local contexts.

## Background

Tobacco-use and exposure to air pollution (AP) are two major risk factors for non-communicable diseases (NCDs), accounting for nearly 30% of the world’s NCD burden [1, 2]. Low- and middle-income countries (LMICs) and vulnerable populations in high-income countries are often disproportionately affected by both the risk factor exposure and subsequent disease burden [3, 4]. Exposure to AP (ambient and household) is the topmost contributor to NCD burden, estimated to account for 6.7 million deaths globally, with nearly 90% occurring in LMICs [5–8]. Tobacco use is the third-leading risk factor responsible for over 8 million deaths a year globally, including 1.3 million non-users who are exposed to second-hand smoke [6, 9]. Nonetheless, tobacco use remains endemic, with an estimated 22.3% of the world’s population using some form of tobacco and 80% of those living in LMICs [10]. As NCDs develop from prolonged and cumulative risk factor exposure, primary prevention early in life is a key public health priority globally [11–13].

Several multi-level, evidence-based interventions are recommended for preventing and reducing exposure to tobacco use and AP, yet are rarely implemented in practice [13]. The WHO’s Global Action Plan on NCDs provides a list of 88 ‘best buys’ and other recommended interventions to address key NCD risk factors, from national level policy actions to community and individual level interventions [14]. Adapting interventions with an existing evidence base rather than developing new interventions in new settings reduces the necessary investment of time and resource [15], but even cost-effective interventions are implemented inconsistently worldwide, limiting their benefits [16].

This implementation gap is particularly large in settings that lack political will, resources and capacity, and, importantly, adaptation of global policies and interventions to the specific social, cultural, economic, legal, political and institutional contexts [17, 18]. Evidence for ‘best-buy’ interventions is typically generated in high-income countries, and effects do not always transfer directly when implemented in contexts that are very different in terms of norms, delivery structures, and resources from original settings [19]. Careful selection and adaptation of interventions and implementation strategies tailored to specific contexts is often required in order to increase their potential for adoption, acceptability, and effectiveness [14, 20].

In this paper, we distinguish three related but separate activities. Intervention selection refers to choosing the intervention or combination of interventions most appropriate for the priority risk factor, target population, setting, and context. Intervention adaptation refers to intentional modification(s) of an evidence-informed intervention to achieve a better fit between the intervention and a new context. Implementation-strategy development refers to identifying and tailoring the actions needed to support adoption, delivery, and sustainment of the selected intervention. Although these activities are defined as distinct, they are far from being linear and are typically interdependent because judgements about suitability often depend on whether an intervention is deemed adaptable and if feasible and acceptable implementation strategies could be developed. Selection, adaptation, and implementation-strategy development all require deliberative decision-making based on judgements about the interventions’ relevance, feasibility, acceptability, scalability, adaptation needs and likely value-for-money [15]. To ensure that these decisions result in an improved fit between the intervention and the new context, it is important that researchers work collaboratively with all relevant stakeholders using a co-creative approach. Stakeholders, broadly defined as all those who are affected by or can influence the implementation of the intervention, are experts in their local context with lived experience of the risk factors, their drivers, the health and social systems and/or the prevailing social and cultural norms, and are therefore best placed to assess the contextual fit of interventions [21]. Co-creation is increasingly advocated in public health intervention research (from development, adaptation to implementation) as a proactive and efficient approach to increasing the contextual-fit, adherence/adoption, acceptability and effectiveness of interventions [22, 23].

**Fig. 1:**
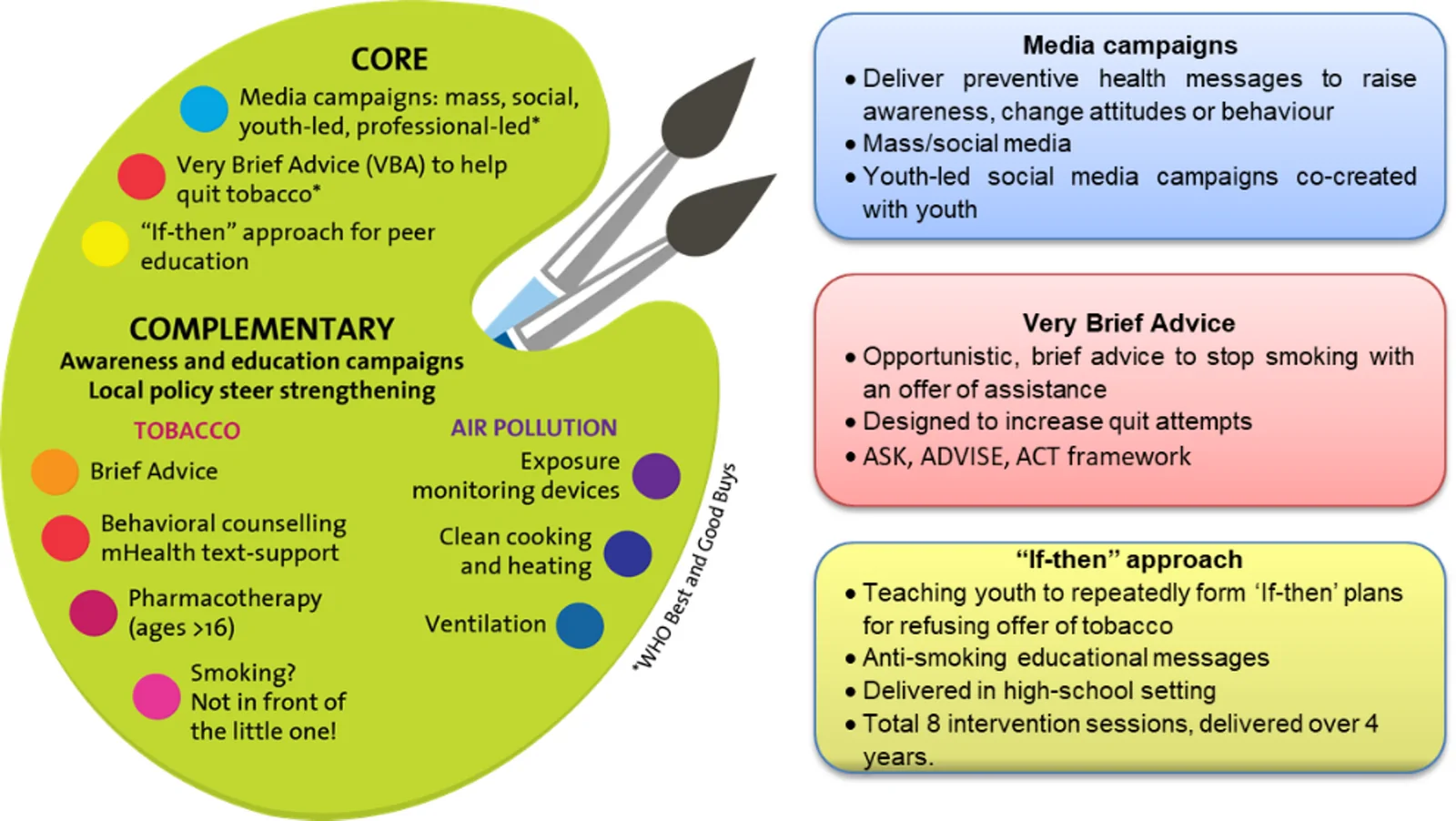
FRESHAIR4Life prevention palette [24] and core intervention description. (Reproduced with permission from Hoffman et al. 2024 [25])

**Fig. 2:**
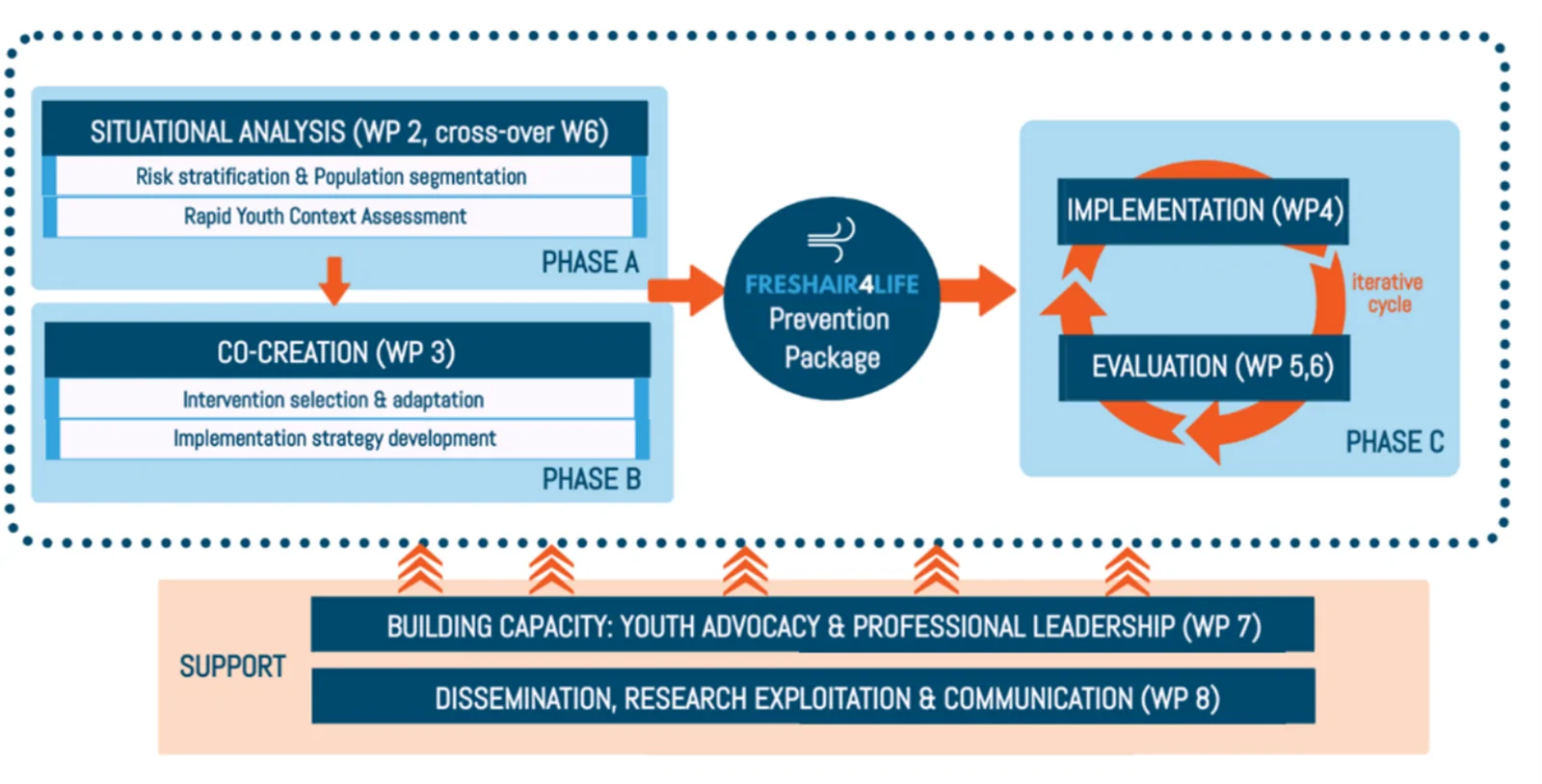
FRESHAIR4Life overall project plan. (Reproduced with permission from Hoffman et al. 2024 [25]). ^1^ Phase 1 Situational analysis, using mixed methods rapid youth context assessment, to understand the perceived burden, the contextual drivers, determinants of behaviour related to risk factors and dynamics among different stakeholders. Phase 2: Co-creation of prevention packages through selection and adaptation of interventions and development of context-specific implementation strategies. Phase 3: Implementation and evaluation of selected interventions using the RE-AIM framework and cost-effectiveness analysis

Recognising the implementation gap and adaptation need in populations with the greatest risk factor exposure, the FRESHAIR4Life project was established in five diverse countries (Greece, Kyrgyzstan, Pakistan, Romania and Uganda) to develop processes for co-designing optimal implementation of tailored, evidence-based interventions for reducing exposure to tobacco and AP among adolescents [24]. The project focussed specifically on adolescents as a crucial developmental stage when health-related motivations and behaviours are formed, offering a window of opportunity to foster healthy behaviours and shape future health. The countries were selected because of their high NCD burden and risk factor exposure and the diversity of their contexts, which offered an opportunity to develop implementation knowledge that could be extrapolated to settings worldwide. In this four-year implementation study, a bespoke ‘prevention palette’ was developed listing (cost) effective interventions based on the WHO best buys [14], the Framework Convention on Tobacco Control MPOWER measures [25], the WHO global action plan [13] for prevention and control of NCDs and relevant literature [26]. Core interventions in the palette included a media campaign, very brief advice (VBA) and a behavioural intervention ‘implementation intentions’ (If-Then approach), which could be complemented with additional strategies based on local burden and context needs (Fig. 1). Local country teams, responsible for the research and implementation, were supported to identify target populations and settings, and select, adapt, implement and evaluate the interventions and implementation strategies. The methodology is detailed in Hoffman et al. [24], but briefly, it comprised three phases: a situational analysis, selection and co-creation of prevention packages and implementation and evaluation of selected interventions (Fig. 2). These broadly align with the steps outlined in ADAPT guidance for adapting and transferring interventions to new contexts by Moore et al. [15].

In this paper we describe the co-creation process used in Phase 2 to select and adapt interventions from the prevention palette and develop implementation strategies tailored to each country’s unique social and cultural context. We discuss the challenges encountered during this process and how they were overcome and offer insights for future implementation research on context-specific NCD prevention in public health.

## Methods

As no published guidance was available on how to select and adapt interventions to reduce adolescent exposure to tobacco and air pollution across diverse contexts, we developed a customised approach. This drew on principles of co-creation/participatory action research [27, 28], decision support, and elements of the Socio-Technical Allocation of Resources (STAR) framework [29]. As full STAR implementation can be a resource-intensive, multi-stage process typically taking 12–16 weeks [30], we pragmatically adapted selected STAR-informed criteria rather than implementing the full STAR process. Specifically, STAR informed the use of structured, criteria-based deliberation, including elicitation of stakeholder judgements about likely reach, benefit, feasibility, acceptability, cost and value, and explicit discussion of assumptions and trade-offs. This provided a structure that combined technical analysis of best available data with stakeholder engagement leading to systematic, transparent decision-making and priority setting based on value. We used ‘stakeholder workshops’ as a methodological tool to enable systematic deliberation and elicitation of local knowledge, ensuring contextual grounding and local ownership.

**Fig. 3:**
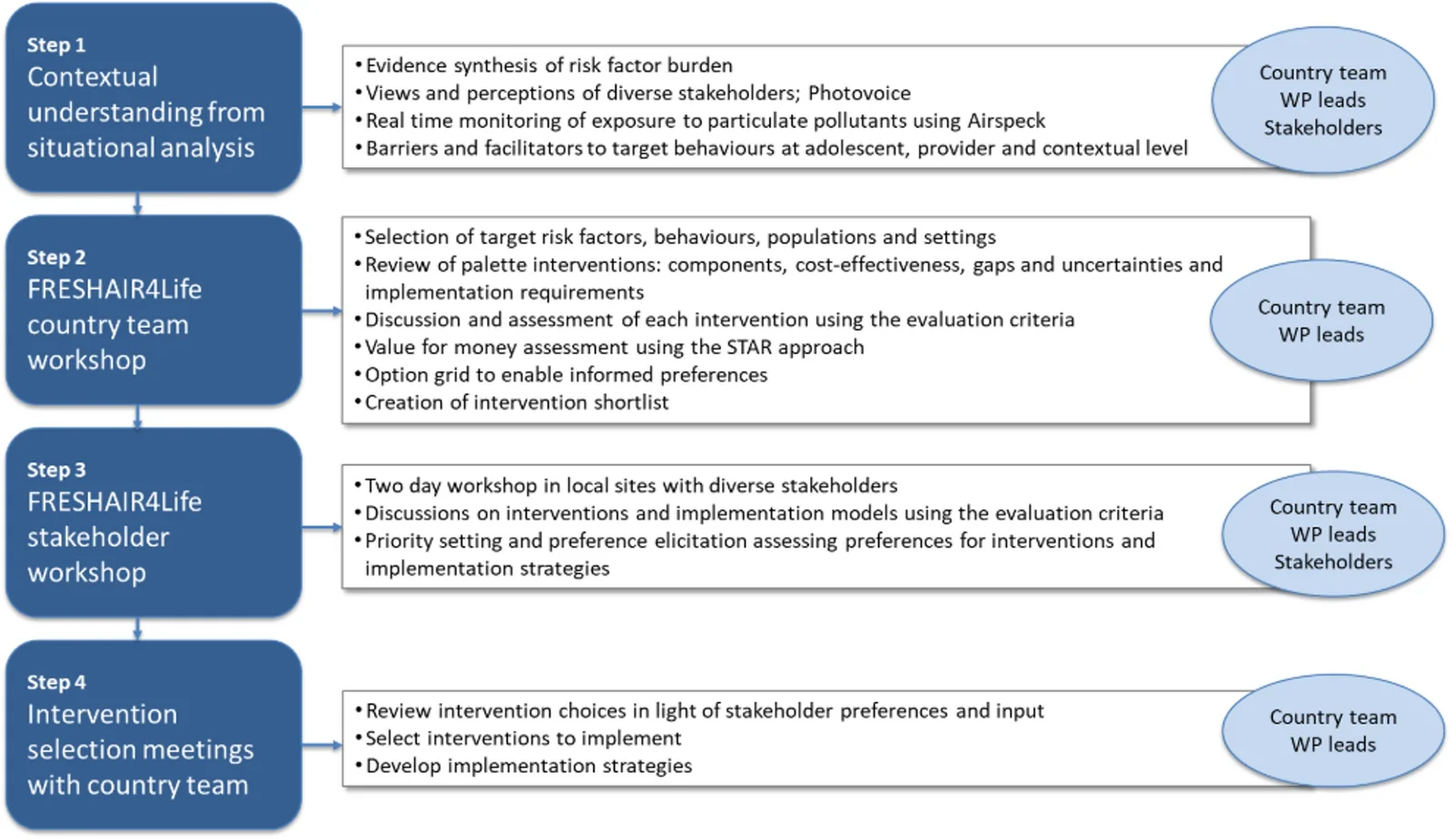
Steps in the process of selection and adaptation of interventions and implementation strategies

In each country, selection and adaptation of interventions and implementation strategies took place in four distinct steps (Fig. 3). The country research and implementation teams (referred to as ‘country team’ hereafter) were supported by the relevant work-package (WP) leads within the FRESHAIR4Life consortium in stakeholder engagement, priority setting, intervention adaptation, implementation strategy development and final selection decisions. Sequential engagement in this process allowed learning from one country’s experience to inform adaptations in subsequent countries. Ethical approval was sought from the research ethics committees in all included countries.

### Step 1: Consolidation of contextual understanding from the situational analysis

The situational analysis [24] completed in each country in Phase 1 involved the collection of qualitative and quantitative data from a variety of sources (desktop reviews, adolescent, healthcare provider and teacher surveys and focus groups, photovoice with adolescents, and real-time monitoring of adolescents’ personal exposure to AP using AirSpeck monitors [31, 32] to understand the actual and perceived burden of tobacco and AP as NCD risk factors and the drivers and determinants of risky behaviours. Rapid analysis and triangulation of these data were used to consolidate the contextual information about each country. Specifically, the barriers and facilitators to target behaviours within each risk factor were identified and classified into three levels: individual level (what influences adolescents’ behaviours in relation to risk factors), social and community level (what influences the support behaviours of adults around adolescents, i.e., service providers) and contextual level (what in the wider socio-political-economic context influences the target and support behaviours). We mapped these factors to constructs from psychological theory using the Theoretical Domains Framework [33] to identify targets for change.

### Step 2: FRESHAIR4Life country team workshop

In each country, the country team (see Table 1 for team composition) participated in two half day online workshops to select risk factors (AP, tobacco use, or both), target behaviours (tobacco prevention/cessation, AP exposure/emission reduction), populations (age/gender) and settings for implementation (school/university/sports clubs/primary care) and to generate a shortlist of interventions from the FRESHAIR4Life palette that they deemed important, feasible, and acceptable in their local contexts. The workshop was structured as follows:

**Table 1:** Composition of the stakeholder workshop in each country

Country	Number of Stakeholders	Stakeholder composition	Country team composition
Kyrgyzstan	19	High school (*n* = 3) and university students (*n* = 12), teachers (*n* = 2), GPs (*n* = 2)	Respiratory medicine specialists and researchers (*n* = 6); respiratory medicine professor and researcher (*n* = 1)
Pakistan	19	Football club students (*n* = 7), youth ambassadors (*n* = 3) coaches (*n* = 2), club manager (*n* = 1), psychologist and health workers (*n* = 3), policy makers (*n* = 1), NGO and community representatives (*n* = 2)	Public health consultant (*n* = 1), pulmonologist (*n* = 1), researchers (*n* = 3), communications manager (*n* = 1), communications assistant (*n* = 1), project manager (*n* = 1)
Greece	27	High school (*n* = 6) and university students (*n* = 4), middle and high school teachers (*n* = 5), nursing fellows (*n* = 3), GPs (*n* = 3), policy makers (*n* = 4), clinician/academics (*n* = 2)	Professor of General Medicine and Public Health (*n* = 1); Assistant Professor of Primary Care and Public Health; nurse and postdoctoral researcher (*n* = 1); general practitioner resident and researcher (*n* = 1); social worker and researcher (*n* = 1)
Uganda	15	High school students (*n* = 9), teachers and school nurses (*n* = 5), policy makers (*n* = 1)	Pulmonologist (*n* = 2), Researchers (*n* = 2), research assistants (*n* = 2), communication specialist (*n* = 1)
Romania	50	High school students (*n* = 25), school counsellors and teachers (*n* = 12), psychologists (*n* = 2), medical providers (*n* = 7), policy makers (*n* = 2)	Family medicine practitioners and researchers (*n* = 3); family medicine professor and researcher (*n* = 1), psychologists (*n* = 3)

- A brief summary of situational analysis findings was presented, including country-specific evidence on risk factor prevalence, disease burden attributable to these risk factors, stakeholder perceptions, and barriers and facilitators to target behaviours at individual, social and community and contextual level. Team discussions focused on selecting risk factors, target behaviours, target populations and settings for implementing interventions.
- Detailed information about each intervention from the FRESHAIR4Life palette was summarised by the International Primary Care Respiratory Group [34] during project set up. This included information on each intervention’s underlying theory, core components, evidence of effectiveness, the context in which evidence was generated, gaps or uncertainties in the evidence, and key considerations for implementation and adaptation.
  Country-specific estimates of the burden of tobacco and AP were presented along with the potential value of palette interventions based on literature. Using online interactive tools that enabled real-time participation (e.g. Mentimeter) and informed by the STAR approach, we elicited estimates of each intervention’s likely reach, short and long-term effectiveness, equity and population-level benefits and costs of both the intervention and the resources used to execute the implementation strategy in the local context. The team also debated the need for each intervention and the feasibility, acceptability, implementation potential, adaptation needs, fit with routine practice and transferability in their context. These discussions did not use the full STAR approach, but used selected STAR-informed criteria to support transparent comparison of intervention options. Together, these evaluation criteria informed our understanding of the overall value of each intervention within the local context.
- Following discussions and deliberations, the teams generated a shortlist of candidate interventions. To prompt deliberation about the implementation and adaptation requirements of the shortlisted interventions and support preference formation, we used an ‘option grid’ approach, which draws on principles of decision science and has been used to support patients to make informed decisions about treatments jointly with health professionals [35]. The grid, when completed with all information, presents a summary of options organised in a tabular format, with options in columns and their key attributes in rows, allowing horizontal comparisons of important and focussed attributes [35]. A blank option grid template was presented to the teams during the second day of the workshop and was used to structure their deliberations on interventions. Subsequently, through internal team meetings, the teams completed the option grid for shortlisted interventions with information on the intervention goal, implementation goal, implementation strategies, material and resource needs, adaptation needs, frequency and duration, personnel and stakeholder requirements, anticipated barriers and facilitators. This prompted the teams to discuss and consider various aspects involved in implementation, which facilitated their assessments of feasibility. Following comparisons among interventions on these attributes, each member independently rated the interventions on a selection of evaluation criteria: likely benefit and reach, importance, long term impact, ease of implementation, cost, and acceptability to adolescents and providers and ranked the interventions in order of preference. The teams mutually finalised the intervention shortlist for selected risk factors (up to 4 interventions) that would be presented to the wider stakeholders for their input.
- Finally, the team members suggested potential stakeholders to invite to the FRESHAIR4Life stakeholder workshop. This was complemented by the stakeholder analysis and mapping exercise completed by the teams during Phase 1, with support and training from the FRESHAIR4Life consortia.

### Step 3: FRESHAIR4Life stakeholder workshop

**Fig. 4:**
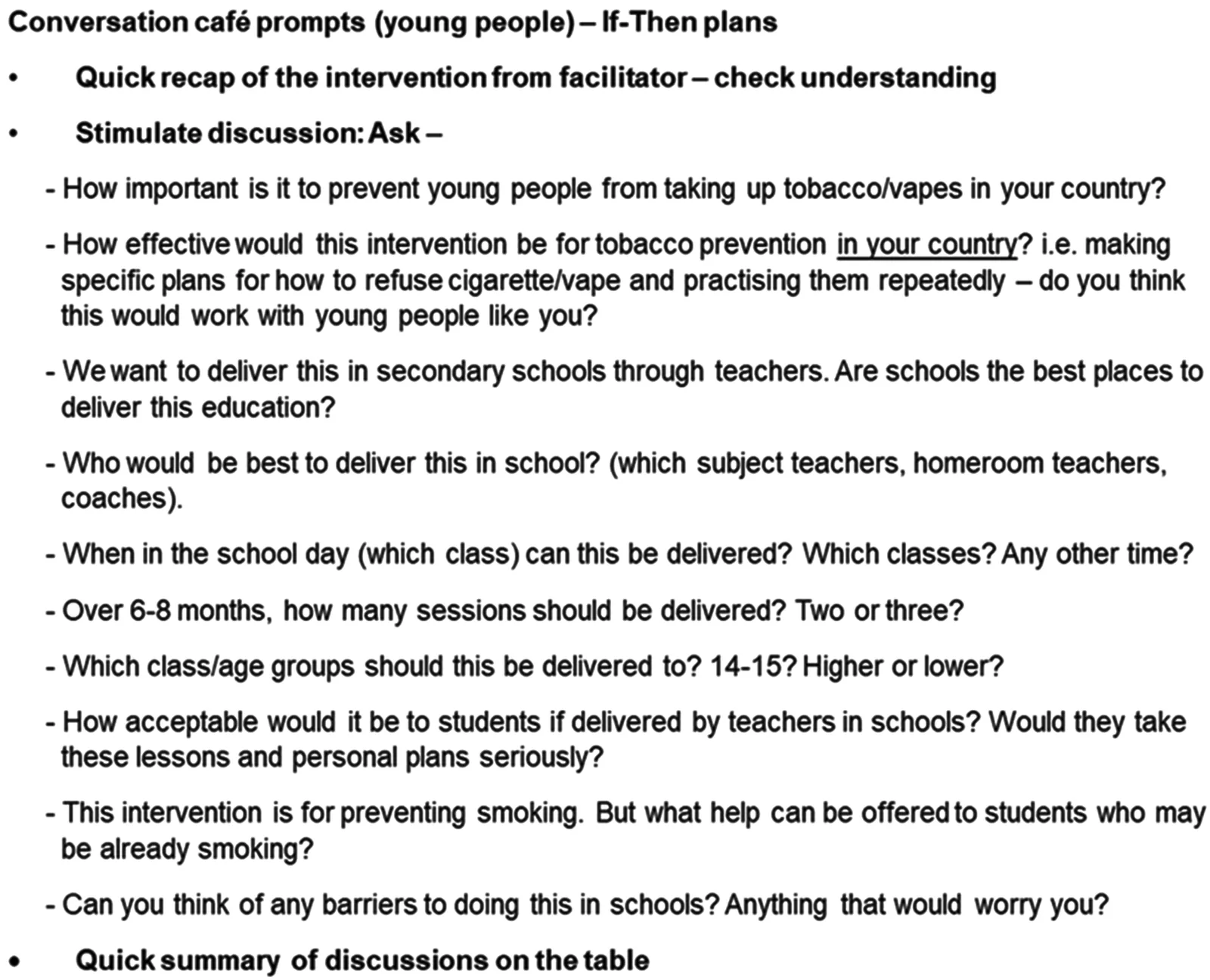
Sample prompt questions guide used in Conversation Cafés during stakeholder workshop

The in-person stakeholder workshop was held in each country in cities or towns that were selected by country teams as implementation sites. The workshop was held over two half days at an accessible venue and was attended by a range of stakeholders including adolescents from secondary schools and universities, education providers, health care providers, policy makers, third sector stakeholders and health researchers and experts (Table 1 for stakeholder composition). The workshop was structured as follows:

- After introducing participants to the FRESHAIR4Life project, we highlighted the burden of tobacco use and/or AP in the country and presented the rationale for focusing on intervening during adolescence. We then provided a brief summary of the stakeholders’ perceptions, barriers and facilitators at different levels, from the situational analysis.
- The shortlisted interventions were presented with their underpinning principles and core components, followed by different proposed models of implementation in different settings (school, healthcare) and target populations (younger vs. older adolescents) within their specific context.
- A participatory action-research approach was adopted using a Conversation Café to engage the stakeholders in discussions around the proposed interventions. The café approach has been found effective for creating an informal and conversational environment, facilitating constructive engagement around complex issues and hosting interactive dialogues with large groups [36, 37]. Stakeholders were divided into small groups of five to seven individuals, seated in a circle to facilitate face-to-face interaction. Adolescents were assigned to separate groups from adults to minimise the influence of power differentials and enable all participants to contribute meaningfully to the discussion. Each group was facilitated by a researcher from the country team using prompt questions (Fig. 4) designed to stimulate a discussion on the need/importance of the intervention, feasibility, acceptability across stakeholder groups, and ideas on adaptation and implementation strategies that could be made to work successfully in their context. Detailed notes were captured by a notetaker and/or from audio recordings made with permission, which were subsequently deleted.
- Following the facilitated discussions, we used a culturally tailored process to elicit stakeholders’ priorities and preferences for interventions and implementation strategies based on the evaluation criteria. This process ensured all stakeholders could voice their views. First, participants estimated the proportion of the target population likely to be reached and benefit from each of the proposed interventions; where relevant, estimates were stratified by age group and gender. Second, participants rated key aspects of each intervention, including importance, likely reach and benefit, immediate and long-term impact, ease of implementation, cost, and acceptability to adolescents and providers, using a Likert scale. Lastly, priorities were elicited either through a fixed budget allocation exercise (e.g., a ‘100 dollar’ exercise used in Kyrgyzstan and Romania), or, where monetary framing was not suitable, through rank ordering (used in Pakistan, Greece, and Uganda). When multiple implementation strategies were presented (e.g. options for who will deliver, when to deliver, which age group to target), these were ranked in the same way. This procedure enabled participants to articulate their value judgments and provide explicit rationales for their preferences, resulting in a defensible, stakeholder-owned ordering of interventions and implementation strategies tailored to the local context. Where possible, these responses were presented separately for adolescents and adults to ensure that the views of both groups were visible except in Pakistan, where it was not possible as all responses were anonymous.

### Step 4: Intervention selection meetings

All the qualitative and quantitative information gathered during Steps 2 and 3, which reflected the views of the country team members and diverse groups of stakeholders, was triangulated and synthesised into a brief report. The quantitative data on ratings and rankings were summarised descriptively and presented graphically. The qualitative notes taken by the facilitators at different stakeholder groups were collated, compared and narratively synthesised noting commonalities and differences across groups and nuanced detail that would be useful in decision making. The report highlighted the collective preferences for target populations, settings, risk behaviours, interventions and implementation strategies; areas where views concurred or diverged; likely barriers, facilitators and challenges to intervention implementation and acceptability; and required adaptations to suit the local context. This summary was discussed with the country team in intervention selection meetings, in which the team revisited their preferences on interventions and implementation strategies in light of all (divergent or concurrent) stakeholder input and reached consensus on the intervention package to implement, feasible implementation strategies, likely adaptations and their fit with routine practice. The ultimate decision was made by the country teams using consensus, with decision-making guidance and support from the work-package leads.

### Reflexivity

This project involved multiple research teams working in different roles. At the country level, the work was led by local teams of professionals (both male and female) including respiratory medicine specialists, clinical academics and practitioners in primary care or family medicine, public health specialists, health researchers, and communication specialists who were participants in the country workshop as well as facilitators of the stakeholder workshop (see Table 1 for team composition in each country). At the FRESHAIR4Life consortium level, this work was led by work-package teams involving three researchers (Indian, American and Dutch) with expertise in health psychology, implementation science, and health economics, who led the country workshops, guided the stakeholder workshops, synthesised workshop outputs and facilitated selection meetings. The diversity in their cultural backgrounds helped establish cultural sensitivity in their approach, overcome each other’s biases, and tailor communication strategies to suit different cultural norms. They were supported by a wider team of implementation researchers, senior academics and experts. Reflexivity was practiced in several ways. The professional status of country team members, often affiliated with health services or research institutions, may have created power imbalances with certain stakeholder groups regarding age, socio-economic status, and authority. To address this, workshops were conducted in neutral venues like hotels or small conference rooms, and younger team members facilitated adolescent groups to improve rapport. Open and critical discussions between country teams and work-package leads ensured that prompt questions in conversation cafes were culturally relevant and aligned with objectives for stakeholder input. Following each stakeholder workshop, debriefing sessions were conducted between country and work-package teams. These sessions provided opportunities for facilitators to share observations and impressions, and for all members to make explicit and challenge their preconceptions, assumptions, and biases, which were noted in the workshop notes. During the synthesis of stakeholder input and selection meetings, work-package leads continuously evaluated their assumptions and biases to avoid unduly influencing the decision-making process.

## Results

Greece, Pakistan and Romania selected the ‘If-Then’ approach for tobacco prevention, while Kyrgyzstan selected VBA and behavioural counselling (VBA+) for tobacco cessation. Pakistan and Uganda opted for a social media campaign to raise awareness of AP. The If-Then approach for tobacco prevention consisted of anti-tobacco educational messages and personal If-Then plans for refusing an offer of tobacco/vape, packaged within the INTENT program [38]. As in our previous FRESHAIR work [39], we defined VBA + as opportunistic, brief advice to stop smoking followed by an offer of support (from a trained behavioural counsellor if available). Social media campaigns – more practical than the evidence-based mass-media campaigns - involved disseminating audio-visual preventive messages through popular social media channels. Outcomes from each of the four steps of the selection and adaptation process in each country are detailed in Table 2. The rationales for intervention selections in each country are outlined in Table 3. The columns show how the selection process unfolded in each country; the rows indicate the similarities and differences in outcomes across the countries at each step. Below, we report the challenges encountered during the process of selection, adaptation and development of implementation strategies along with how they were overcome.

**Table 2:** Outcomes from the four steps of the selection and adaptation process in each country

	Greece	Romania	Kyrgyzstan		Pakistan	Uganda
**Step 1: Main takeaways from the situational analysis**						
Most significant risk factor	Tobacco use	Tobacco use	Tobacco use AP		Tobacco use AP	AP
Drivers of tobacco use	• *Individual level*: Poor knowledge of the specific health consequences of traditional and alternative products, inadequate awareness of cessation methods, inaccurate beliefs about smoking and vaping, boredom and stress, social pressures, identity-related influences. • *Social & community level*: Lack of knowledge and skills among providers in prevention and cessation among adolescents, a sense of helplessness and lack of opportunities to help. • *Contextual level*: Weak enforcement of tobacco-related laws and regulations					
Drivers of air pollution				• *Individual level*: Poor knowledge about the health consequences of AP, low perceived susceptibility to AP’s effects, low perceived severity of AP effects, low salience and priority of AP, lack of knowledge on reducing exposure, low perceived control over exposure, sense of helplessness, low level of concern • *Social and community level*: Little knowledge of solutions, lack of education about AP, low confidence, limited skills & opportunities to teach about AP • *Contextual level*: Pervasiveness of AP, lack of rule enforcement, old polluting cars, no curriculum on AP, low political priority, high poverty linked to exposure and emissions, low affordability of clean fuels/technologies.		
**Step 2: Initial preferences and proposals by country teams**						
Selected Risk factor	Tobacco use	Tobacco use	Tobacco use Air pollution		Tobacco use Air pollution	Air pollution but also diverse exposures
Target behaviour	Tobacco use prevention; tobacco cessation	Primary: tobacco use prevention; secondary: tobacco cessation	Tobacco use prevention; tobacco cessation. AP exposure reduction		Tobacco use prevention; AP exposure reduction	AP exposure reduction
Target Population	12–15 year olds for prevention 18–21 year olds for cessation Urban	14–15 year olds Urban	14–21 year olds Urban & rural		14–21 year olds Urban, low socio-economic groups	12–18 year olds Urban
Target Setting	High schools, universities	High schools	High schools, family medicine centres		Local football club	High schools
Intervention shortlist for stakeholder workshop	• Implementation intentions (If-then) • Very Brief Advice (VBA)	• Implementation intentions (If-then) • Very Brief Advice (VBA) • Smoke-free school policy	• Implementation intentions (If-then) • Very Brief Advice (VBA) • Social media campaigns		• Implementation intentions (If-then) • Social media campaign for AP	• Fans in classrooms • Face masks/coverings • Brief advice on reducing exposure • Educational campaigns
**Step 3: Stakeholder views**						
Intervention preferences	• Good support for both interventions. • Implementation intentions (if-then) preferred by adolescents over VBA • VBA challenging in schools but possible in university.	• Mixed preferences. • Smoke-free school policy preferred but rated challenging and less acceptable. • Adolescents preferred VBA, but providers preferred Implementation intentions (if-then). • Multiple challenges anticipated for any intervention.	• VBA preferred along with social media campaigns for tobacco and AP. • Little support for Implementation intentions (if-then)		• Good support for both interventions	• Preference for educational campaigns • Moderate support for face masks • Fans in classrooms and brief advice deemed less feasible, acceptable or effective.
**Step 4: Final intervention selections**						
Interventions to implement and evaluate	• Implementation intentions (if-then) + Embedded mechanism for cessation support	• Implementation intentions (if-then) + Embedded mechanism for cessation support	• Very Brief Advice + behavioural counselling • Social media campaign for tobacco		• Implementation intentions (if-then) + Embedded mechanism for cessation support • Social media campaign for AP	• Social media campaign to increase awareness of Ambient AP and personal exposure reduction strategies identified as feasible, such as using face coverings and avoiding busy roads
Population	12–15 year olds	14–15 year olds	14–21 year olds		14–21 year olds	16–18 year olds
Setting	Middle & High school setting	High school setting	Family medicine centre (primary care)		Football club	Social media platforms: Instagram, Tik Tok, X.
Who	Teachers (self-selected)	Teachers (self-selected)	GPs & Feldshers^1^ to deliver VBA Trained nurses to deliver behavioural counselling (self-selected)		Football coaches (self-selected)	Country team, promoted by youth ambassadors (self-selected)
When/how often	2 sessions over 2 school terms	3 sessions over 6 months During “Dirigentie^2^ class”	Opportunistically when adolescents visit clinic for any reason		4 sessions over 8 months	4–6 weeks duration
Adaptations	• Cultural adaptation of content by research team, adolescents and teachers • Train teachers to encourage cessation and sign-post to support.	• Cultural adaptation of content by research team, adolescents and teachers • Train teachers to encourage cessation and sign-post to support	• Use previously adapted VBA training materials		• Cultural adaptation of content by research team. adolescents and coaches • Simplification of content to adolescents’ literacy levels • Adaptation of content to sports context • Adaptation of training materials to coaches’ literacy levels • Train coaches to encourage cessation and sign-post to support	• Creation of Videos, visuals, captions tailored to local context • Adolescent input and feedback on campaign materials • Interactive elements to promote engagement
Implementation strategies	• School information sessions to parents, teachers, and students to inform about implementation • Teachers trained by trained master trainers	• School information sessions to parents, teachers, and students to inform about implementation • Teachers trained by trained master trainers	• 1 day + refresher training by trained master trainers • VBA practice mandated through Ministry of Health • Prepare VBA champions • Desk-side VBA prompts • Tools for quality monitoring		• Information for parents/students • Coaches trained by trained master trainers • Integration of sessions within regular football training	• Identify and train school ambassadors to lead peer engagement and increase campaign visibility

^1^physician assistants trained to provide a wide range of services, often where doctors are scarce

^2^Specific Romanian term for home-room teacher (USA) or form tutor (UK)

**Table 3:** Rationale for intervention selections in each country

Country	Intervention	Rationale for intervention choices
Greece	Implementation intentions (if-then) + Embedded mechanism for cessation support	• Overall consensus among stakeholders and county team that this intervention was important and needed in Greece • It provided a novel approach to tobacco prevention, which was necessary as previous prevention attempts had been unsuccessful. • Adolescents had stronger preference for this intervention as skills in refusing offer of smoke were needed in a society where smoking was the norm and highly prevalent among young people. • Reach of this intervention was considered higher than VBA
Romania	Implementation intentions (if-then) + Embedded mechanism for cessation support	• No intervention received highly positive evaluation from stakeholders, suggesting multiple and diverse challenges in implementation • Prevention of initiation of smoking was considered as highly important by all stakeholders and country team • Despite differences in initial preference among adults and adolescents for VBA and If-then approach (adolescents preferred VBA over If-then plans but adults preferred if-then plans over VBA), both groups finally agreed that if-then would have the greatest reach and benefit among adolescents.
Kyrgyzstan	Very Brief Advice + behavioural counselling	• Strong perceived need among adolescents for offering opportunities for cessation as existing rates of tobacco and nicotine use were considered high among young people. • Despite some scepticism from GPs, adolescents felt that VBA offered a more direct opportunity to work with those who smoke, used a tailored/individualised approach and the credibility of health professionals would enhance likely effectiveness.
	Social media campaign for tobacco	• Considered important by health professionals to raise awareness and knowledge of tobacco and nicotine harms among young people, change their beliefs and norms and motivate them to quit or not initiate smoking. • GPs had concerns about limited reach of VBA and uncertainty of effectiveness in adolescents, thus they favoured an intervention with greater adolescent reach. • No previous campaigns on tobacco in the country.
Pakistan	Implementation intentions (if-then) + Embedded mechanism for cessation support	• Smoking was considered as a significant problem, highly normalised in Pakistani society, with initiation as early as age 11–12. • Previous cessation efforts in the country had poor reach and cost-effectiveness, and prevention efforts were rare. • Stakeholders and country team considered prevention as most important • An established relationship with the local football club and their keen interest in helping to deliver the smoking cessation message.
	Social media campaign for air pollution	• AP considered as a big problem, but less salient and therefore ignored. • Stakeholders wanted to address the misconceptions that AP is more a government responsibility and individuals and communities have little control over it. • Social media campaigns considered effective ways to reach adolescents, raise awareness of the health risks and encourage protective and pro-environmental behaviours.
Uganda	Social media campaign to increase awareness of Ambient AP and personal exposure reduction strategies	• Situational analysis revealed higher exposure among adolescents to ambient AP compared to household AP, greater diversity in type and size of particles, location, duration and timing of exposure and limited adolescent awareness of health risks associated with ambient AP and of the ways to reduce personal exposure. • General lack of evidence-based interventions for reducing personal exposure to ambient AP feasible/appropriate for Ugandan context • Educating on the importance of reducing personal exposure to AP considered essential by all before any other interventions • Social media campaign also preferred by all stakeholders.

### Challenges in selecting and adapting interventions

#### Identifying a sound basis for intervention selection decisions

Selecting interventions from the palette of evidence-based interventions was a complex decision-making process. The complexity arose from the number of potential interventions; the multiple, often conflicting values of the evaluation criteria (e.g. reach, benefit, impact, cost, ease of implementation, feasibility, acceptability); the differing perspectives of stakeholders (e.g. the country teams, adolescents, providers, policy makers); and the need to make difficult trade-offs (e.g. an intervention may be effective but have limited reach in the target population). Because all options were evidence-based, there was no obvious right or wrong decision; the right choice depended on balancing favourable and unfavourable attributes against country-specific needs and context. This meant that the quality of the decision should be determined by the process by which the decision is made, rather than the choice of a particular intervention.

Several information sources underpinned the intervention selection decisions in this project, but each source had its limitations. First, much of the evidence underpinning the interventions was generated in contexts that were significantly different from the countries or age groups in which they were to be implemented. In LMICs in particular, differences in implementation processes can be decisive for outcomes, leaving gaps in our knowledge base. For instance, the lack of evidence for VBA among adolescents, and the lack of interventions for reducing exposure to AP compared to the number of options for tobacco use. The second source of information came from the stakeholder evaluation of the interventions’ evaluation criteria such as need, benefit, reach, acceptability, cost and feasibility. The views of the country team, which stemmed from their academic and professional experiences, and those of the stakeholders, which stemmed from their lived experiences, did not always align. Balancing these different views increased the difficulty of the decision-making process.

This challenge was tackled by different countries in different ways. In Pakistan, the team had a strong pre-existing preference for the ‘If-Then’ prevention intervention. This was due to various reasons including prevention being highlighted as a priority in the situational analysis, very limited previous prevention efforts in the country and keen interest from the local football club for collaboration. As a result, the stakeholder workshop focused on how to best adapt and implement the If-Then intervention so it would be feasible and acceptable in the local context, rather than on eliciting preferences about which interventions to implement. In all other countries, there was more of an equipoise among the teams about which intervention to implement, and stakeholder input was crucial in informing their preferences for interventions as well as decisions on feasible and acceptable implementation strategies.

#### Reaching and helping existing tobacco users while focusing on prevention

The If-Then tobacco prevention intervention targets 12-16-year-olds in high school setting, and is delivered in a total of eight 50 min sessions over four years (two sessions per year). Each session consists of anti-tobacco messages and repeated formation of implementation intentions to refuse an offer of tobacco in the form of an If-Then plan. The original (INTENT) intervention [38], developed in the UK [26], does not include any explicit mechanism for offering cessation support to existing tobacco users; but not offering support to existing tobacco and vape users created an ethical dilemma for the country teams in Greece, Romania and Pakistan where the prevalence of tobacco use among adolescents is high. A further dilemma was the lack of licensed pharmacotherapy to address dependence in this age group.

Supporting existing smokers was further complicated by the lack of evidence-based cessation interventions for adolescents in school settings; there was no evidence that VBA can be implemented effectively in schools. There were many reasons why a school setting was not conducive to implementing VBA such as limited opportunities for 1–1 conversations with students, student’s health not being the expected focus of discussions in educational settings, no provision of further support for cessation in schools (e.g. behavioral counselling, medication), potential sanctions against students who smoke at school, smoking cessation not being viewed as part of teachers’ role and complexities around parental consent and involvement.

Selecting the If-Then approach therefore, required a commitment to considering mechanisms for offering cessation support to existing tobacco users. Teachers and coaches would need training on how to advise and motivate the students to quit tobacco or vaping; offer a listening ear to those who may want to talk about their tobacco use; and direct/refer students to locally available cessation resources, typically including contacting primary care practitioners, school nurse or psychologist, local quitlines or WHO’s AI based virtual health worker Florence (now renamed Sarah) [40].

#### The paucity of interventions for ambient AP

This project focused on selecting evidence-based interventions, which was facilitated by the intervention palette. However, most options in the palette targeted tobacco use rather than AP, and the evidence-based AP interventions predominantly addressed household AP (ventilation and clean fuel) rather than ambient AP. This created a challenge for countries (such as Uganda) where ambient AP was the risk factor they wanted to address.

In Uganda, 99.4% of the population relies on polluting fuels and fuel technologies [41], so household AP was assumed to be a significant risk factor and potential focus for FRESHAIR4Life activities. However, the situational analysis revealed that adolescents experienced higher exposure to ambient than to household AP, including exposure in school classrooms as well as when commuting to school. Personal monitors revealed a huge diversity not only in the type and size of particles but also locations, timing and duration of exposure to AP faced by adolescents in Uganda. The exposures were pervasive and included vehicle emissions, dust, particles from burning biomass/non-biomass, indoor and outdoor. This was complemented by evidence that, in urban settings like Kampala, the ambient AP averaged 10 times the WHO-recommended air quality limits [42, 43]. This posed a challenge in intervention selection given the lack of evidence-based interventions for reducing personal exposure to ambient AP that were relevant and feasible for implementation at the individual adolescent or community level.

A broader search for interventions beyond the FRESHAIR4Life palette revealed very few individual or community level interventions. Recommendations were mainly structural and policy-level (e.g. legislation around distance of schools from polluting sources, cycle paths to school, car-free zones around schools, introduction of green school transport, tree plantations, adopting standards for AP levels in schools) [44], or lacked cultural and contextual relevance, affordability and feasibility (e.g. reducing time spent in polluted outdoor environments, changing location and timing of outdoor physical activity, air cleaners, N90 face masks and active transportation) [45]. The only feasible recommendation was raising awareness of causes and health effects of AP among adolescents and educating them on ways of reducing personal exposure to AP (such as taking quieter roads, wearing close-fitting face coverings, opening windows while cooking). As stakeholders also preferred this approach, the country teams selected an awareness campaign delivered through social-media for implementation.

### Challenges in developing implementation strategies

#### Implementation strategies as an iterative process

Developing implementation strategies was integral to the process of selecting and adapting interventions, and the two were often inter-dependent. Intervention choices depended on the feasibility of proposed implementation strategies, yet assessing the feasibility of strategies required close consultation and collaboration with relevant stakeholders, which often followed intervention selection. As a result, implementation strategy development was an iterative process, evolving as uncertainties about feasibility, practicalities, and availability of resources needed to be addressed.

Kyrgyzstan chose to implement VBA in family medicine centres, however, there were many uncertainties about implementation strategies e.g. how best to recruit practitioners for VBA training and implementation; how to ensure confidentiality when discussing smoking with adolescents who attended clinics with parents; and how to monitor VBA practice. Addressing these aspects needed thorough consultation and preparation with family medicine practices. As new insights emerged on resources, stakeholder buy-in, and feasibility, some plans needed to be abandoned. For instance, the initial plan to train nurses in behavioural support for cessation proved not to be feasible.

#### Embedding the interventions into routine practice

Adoption is more likely when interventions integrate smoothly into existing routines and cause minimal disruption. This largely depends on the opportunities that are available in the context to enable seamless integration. In case of the If-Then intervention, not all contexts had appropriate opportunities to deliver the teaching sessions to the target populations. In UK schools, where the intervention was originally developed, it was integrated within the existing health and well-being curriculum, which had dedicated class time. However, no such curriculum or dedicated class time existed in the educational systems of Romania and Greece. The first step in these contexts was to identify suitable opportunities for intervention delivery. In Romania, the class time of ‘homeroom teachers’ was identified for delivery and in Greece, volunteer teachers incorporated the delivery within their respective classes.

Pakistan selected to implement this intervention in the context of football clubs, where this type of teaching is significantly different from the coaches’ daily practice. Although the sports/fitness context aligned well with teaching to refrain from tobacco, the context was less conducive to formal delivery of this education. For instance, the teaching does not take place in classrooms and the agenda of lessons is ‘active play on the playground’, which meant that the original format of the If-Then sessions did not fit naturally into the daily routine. Delivering the intervention content was challenging for coaches due to varying literacy levels and lack of familiarity with classroom style teaching, which necessitated considerable adaptation to the content and duration of the sessions. This prompted adaptations of the intervention content, sessions, duration and complexity in order to embed the intervention within regular training activities.

## Discussion

Reducing exposure to major NCD risk factors such as tobacco use and AP is an important goal for public health practitioners and authorities worldwide. A broad range of tobacco control interventions have been developed and utilised over the last two decades, though there are concerns that these do not necessarily translate into improved population cessation rates [46]. Selecting and prioritising interventions from the menu of evidence-based solutions can be challenging when there are uncertainties about the evaluation criteria such as feasibility, acceptability, cost-effectiveness and implementability of the interventions within the specific country contexts.

**Fig. 5:**
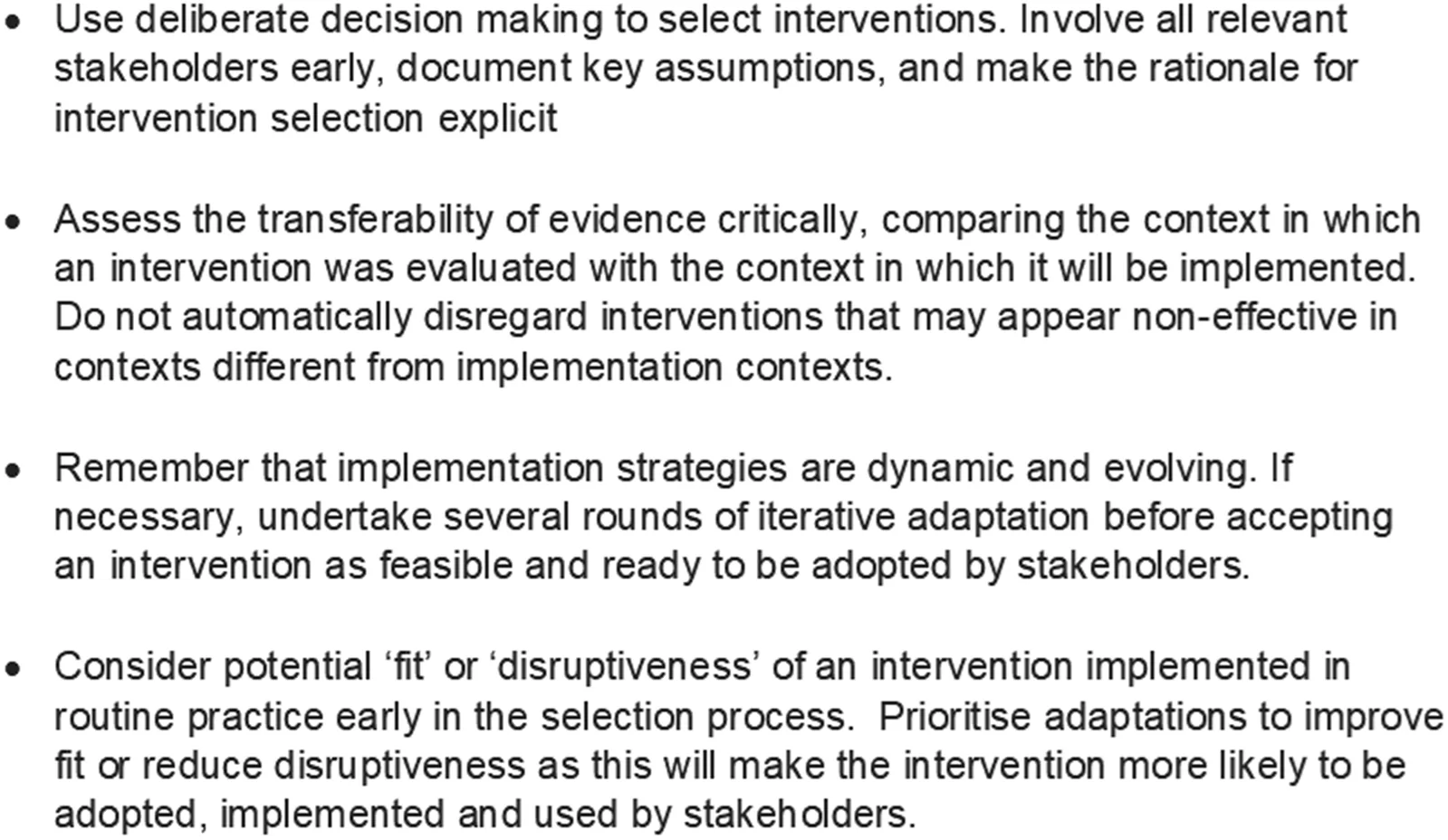
Implications for future implementation research and practice

Tobacco control research in the United States has criticised its ‘uneven utilisation’ of implementation science tools [47]. We used a co-creation approach to select and adapt interventions in five diverse countries within the FRESHAIR4Life project and identified significant challenges with our systematic process. Across five diverse settings, the quality of our intervention choices hinged less on identifying a single best intervention and more on following a transparent, criteria-driven, stakeholder-owned decision process. Where the options from the evidence-based interventions palette did not fit our context or were not present (as in the case of AP), it was necessary to adopt a flexible approach that considered context, delivery opportunities, and feasible implementation strategies. Co-creation with stakeholders provided input on the need, feasibility, acceptability and cost-effectiveness, and implementation, which proved vital for selection decisions. The implementation strategies were co-created with stakeholders in an iterative process, which was needed to address uncertainties as they emerged. Adoption seemed to be strongly influenced by how well the intervention fitted with routine practice, so this should be considered as early as possible. Below, we present critical reflections on our experience of undertaking this process and discuss strengths and limitations. Implications for future implementation efforts and research are presented in Fig. 5.

### Reflections on the ‘prevention palette method’

To facilitate the selection of interventions in each country, we used an innovative prevention palette method. The palette presented a menu of candidate interventions identified from the synthesis of available evidence (including WHO best buys [14], the WHO global action plan [13] for prevention and control of NCDs and relevant literature [26]). While this pre-defined list of interventions served as a useful starting point during the selection process, it sometimes proved restrictive to stakeholders when trying to find an optimal intervention for the needs of their context. For instance, the palette offered limited options for reducing exposure to ambient AP, which Uganda prioritised. Additionally, weak transferability of the evidence generated in high income countries to LMIC also limited choices. For instance, some evidence-based interventions for reducing exposure to ambient AP (e.g. air purifiers) were not feasible in the context of Uganda, whereas interventions that may offer some degree of protection from some of the sources of AP common in LMICs (e.g. face masks to mitigate exposure to dust) were not evidence-based. Lack of policy interventions in the palette also resulted in missed opportunities to tackle important up-stream determinants of risk factors such as enforcing tobacco advertising and promotion bans [48]. For instance, the prevalent tobacco culture in schools in Greece and Romania required a more local policy level approach, such as smoke-free-schools policy, recommended by the WHO but with limited evidence and not available in the palette.

### Reflections on co-creating with adolescents

Adolescents aged 14–21 were key stakeholders across all countries and contributed valuable input to the FRESHAIR4Life stakeholder workshops. Their participation was facilitated by the country teams’ existing or developing links with local schools and sports clubs. Engaging adolescents in topics like tobacco use and AP was thought to be challenging due to the implicit assumption that health-related topics were inherently unappealing to adolescents and socio-cultural norms may limit their participation in workshops involving teachers and health professionals. However, our expectations were surpassed by adolescents in all countries. They expressed concerns about the effect of the risk factors on their future health, endorsed prevention aims, voiced agency in tackling these issues, and shared honest and critical feedback on the acceptability and feasibility of interventions. Actively involving youth in co-design of health interventions has been shown in previous research to enhance their relevance, acceptability and likely effectiveness [49, 50]. We recommend engaging with the youth early by reaching out to schools, universities and other systems working with youth.

### Strengths and limitations

Strengths include our systematic and theory-driven approach to selection of interventions drawing on the principles of STAR method, participatory action research and decision support. We used both qualitative and quantitative approaches to consult stakeholder views and preferences. Our staggered approach helped us refine the process and methods used as well as carry over the learning from one country to another. Finally, our approach enabled meaningful engagement with young people on sensitive topics by mitigating power dynamics, assuring confidentiality, using youth-centric and culturally sensitive language and overcoming practical barriers to engagement (e.g. transport, school permission).

A common limitation across sites was the under-representation of adolescents who were users of tobacco or alternative products. We did not ask for their smoking status, but it was clear from the discussion that all the groups included current or former smokers as well as non-smokers, some of whom claimed to be speaking for their smoking friends. This was probably of little consequence for the discussion on tobacco prevention, but it may have resulted in some imbalance in perspectives when discussing VBA though this is difficult to assess as we were not certain of representation in each group. Another limitation was the lack of intervention options for AP and lack of policy interventions in the palette. Finally, the whole process, while rigorous and systematic, was time and resource-intensive. The value of engaging in this process must be balanced with availability of financial and human resources. However, adoption of the core elements such as understanding of context, stakeholder partnership, and deliberative decision-making based on qualitative and quantitative inputs would be valuable.

One of the biggest adaptation decisions concerned the WHO Best Buy “mass media campaign” intervention on the palette. It emerged from early stakeholder engagement including with the country team members that the budgets were not sufficient for a mass media campaign. Furthermore, the situational analysis revealed that mass media would not reach the target adolescents. Therefore, asocial-media campaign was substituted as an intervention despite weak evidence for its effectiveness in creating behaviour change [51, 52]. This represented a pragmatic choice in the absence of stronger alternatives. The choice was supported by several factors, including the potential to reach adolescents, low financial cost, and a realistic focus on raising awareness rather than directly changing behaviour. An important limitation that flows from this choice was the competence of the research consortium in social media campaigns. Therefore, a new capacity building strategy was added. This included the appointment of a communications lead(s) from each country team and from key work packages, who were coached in the development of such campaigns by an experienced communications team.

### Implications

Selecting feasible, acceptable, and cost-effective interventions and adapting to implement in local contexts is an important step in implementation research. We recommend engaging in a systematic and transparent decision-making process documenting various assumptions and discussions with all relevant stakeholders to make defensible choices.

The palette method proved useful in facilitating selection of interventions, however, we recommend that it is used flexibly so that additional context-relevant interventions could be considered. We also recommend critically appraising both the strength of the evidence and the settings in which it was generated. Consistent with the ADAPT guidance, interventions that do not reach the threshold for conclusion of effectiveness should not automatically be disregarded, as ‘non-effectiveness’ could be a result of differences in implementation strategies or context. This is particularly important where cultural, contextual, systemic, capacity and resource differences are particularly stark, often shaping outcomes more than intervention efficacy itself. Policy interventions should also be included in the palette to provide opportunities for implementing systemic changes.

Implementation strategies are dynamic and evolving. They may need considerable adaptation before an intervention can be deemed feasible and acceptable and therefore selected.

It is important to consider ‘fit’ or ‘disruptiveness’ of intervention to routine practice early in the process. Adaptations to improve fit and reduce disruptiveness are more likely to be adopted, implemented and accepted by stakeholders.

Finally, there is still a great need for evidence on interventions to reduce personal exposure to AP, especially in the context of LMICs. Future research should focus on testing available interventions in the specific contexts they are to be implemented or developing contextually tailored solutions.

## Conclusion

Our experience in FRESHAIR4Life shows that, across diverse country contexts, defensible intervention selection depends not only on what is chosen, but on how choices are made. Public health researchers, practitioners and policy makers should use an iterative, transparent and systematic decision-making process involving all relevant stakeholders. Menus of evidence-based interventions should be used flexibly, scrutinising the transferability of underpinning evidence and documenting assumptions and trade-offs explicitly. Stakeholders should be engaged early to assess and improve contextual fit through adaptations and implementation strategies. Early considerations of how interventions can fit within routine and any ethical obligations will help increase the odds of adoption and sustainable implementation. Our experience also highlights an urgent need for research investment in developing feasible individual and community level interventions for reducing adolescents’ personal exposure to ambient air pollution in LMIC settings.

## Supplementary Information

Below is the link to the electronic supplementary material.

Supplementary Material 1

## Data Availability

No datasets were generated or analysed during the current study.

## Abbreviations

AP: AP
NCD: Non-Communicable Diseases
VBA: Very Brief Advice
LMIC: Low and middle income countries
WHO: World Health Organisation
STAR: Socio-Technical Allocation of Resources
FCTC MPower: Framework Convention on Tobacco Control. Monitoring tobacco use, Protecting people from tobacco smoke, Offering help to quit tobacco, Warning about dangers of tobacco, Enforcing tobacco advertising, promotion and sponsorship bans, and Raising taxes on tobacco
