# Supplementary material for: A co-creation approach to selecting and adapting adolescent tobacco and air-pollution interventions and strategies for implementation in diverse countries: insights from FRESHAIR4Life
Source: Implement Sci Commun. 2026 Jul 20;7:188. doi: 10.1186/s43058-026-01044-4 (PMC13584592; doi:10.1186/s43058-026-01044-4)
Supplement: Supplementary file 1 [file 43058_2026_1044_MOESM1_ESM.docx]

Supplementary Figure 1: Stakeholder workshop estimates of intervention reach and benefit by country, stakeholder group, and intervention

*Pakistan data are shown overall because fully anonymous data collection precluded stratification by stakeholder group (adolescents or adults).
